# *N*-Carbethoxy-*N*′-[3-(4-methylphenyl)-1*H*-1,2,4-triazol-5-yl]thiourea

**DOI:** 10.1107/S1600536810004289

**Published:** 2010-02-06

**Authors:** Anton V. Dolzhenko, Geok Kheng Tan, Lip Lin Koh, Anna V. Dolzhenko, Wai Keung Chui

**Affiliations:** a Department of Pharmacy, Faculty of Science, National University of Singapore, 18 Science Drive 4, Singapore 117543, Singapore; b Department of Chemistry, Faculty of Science, National University of Singapore, 3 Science Drive 3, Singapore 117543, Singapore

## Abstract

The title compound, [systematic name: ethyl ({[3-(4-methylphenyl)-1*H*-1,2,4-triazol-5-yl]amino}carbonothioyl)carbamate], C_13_H_16_N_5_O_2_S, exists in the 3-aryl-5-thio­ureido-1*H*-1,2,4-triazole tautomeric form. The mol­ecular structure is stabilized by intra­molecular hydrogen bonding (N—H⋯S=C between the endocyclic N-bound H atom and the thio­ureido S atom, and N—H⋯O=C within the ethoxy­carbonyl­thio­urea unit), both arranged in an *S*(6) graph-set motif. The mean planes of the phenyl and 1,2,4-triazole rings make a dihedral angle of 6.59 (10)°. In the crystal structure, the mol­ecules form two types of centrosymmetric dimers connected by inter­molecular hydrogen bonds; in the first, the N—NH triazole sides of two mol­ecules are connected [*R* ^2^_2_(6) graph-set motif] and the second is an N—H⋯S=C inter­action between the imide H atoms and the thio­carbonyl S atoms [*R* ^2^_2_(8) graph-set motif]. Together, they form a network parallel to the (111) plane.

## Related literature

For the synthesis, tautomerism and structures of related 1,2,4-triazoles, see: Dolzhenko *et al.* (2009*a* ▶,*b* ▶,*c* ▶, 2010 ▶); Buzykin *et al.* (2006 ▶). For related carbethoxy­thio­ureas, see: Dolzhenko *et al.* (2010 ▶); Huang *et al.* (2009 ▶); Lin *et al.* (2004 ▶, 2007 ▶); Su *et al.* (2006 ▶); Zhang *et al.* (2003 ▶, 2007 ▶). For the graph-set analysis of hydrogen bonding, see: Bernstein *et al.* (1995 ▶).
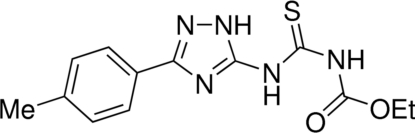


## Experimental

### 

#### Crystal data

C_13_H_15_N_5_O_2_S
*M* *_r_* = 305.36
Triclinic, 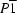

*a* = 6.8430 (5) Å
*b* = 8.7789 (6) Å
*c* = 12.2563 (9) Å
α = 90.780 (1)°
β = 99.425 (1)°
γ = 101.279 (1)°
*V* = 711.52 (9) Å^3^
*Z* = 2
Mo *K*α radiation
μ = 0.24 mm^−1^
*T* = 100 K
0.56 × 0.46 × 0.24 mm

#### Data collection

Bruker SMART APEX CCD diffractometer
Absorption correction: multi-scan (*SADABS*; Sheldrick, 2001 ▶) *T*_min_ = 0.877, *T*_max_ = 0.945
9015 measured reflections
3243 independent reflections
3088 reflections with *I* > 2σ(*I*)
*R*_int_ = 0.021

#### Refinement

*R*[*F* ^2^ > 2σ(*F* ^2^)] = 0.036
*wR*(*F* ^2^) = 0.095
*S* = 1.07
3243 reflections
204 parameters
H atoms treated by a mixture of independent and constrained refinement
Δρ_max_ = 0.44 e Å^−3^
Δρ_min_ = −0.31 e Å^−3^

### 

Data collection: *SMART* (Bruker, 2001 ▶); cell refinement: *SAINT* (Bruker, 2001 ▶); data reduction: *SAINT*; program(s) used to solve structure: *SHELXS97* (Sheldrick, 2008 ▶); program(s) used to refine structure: *SHELXL97* (Sheldrick, 2008 ▶); molecular graphics: *SHELXTL* (Sheldrick, 2008 ▶); software used to prepare material for publication: *SHELXTL*.

## Supplementary Material

Crystal structure: contains datablocks I, global. DOI: 10.1107/S1600536810004289/kp2249sup1.cif

Structure factors: contains datablocks I. DOI: 10.1107/S1600536810004289/kp2249Isup2.hkl

Additional supplementary materials:  crystallographic information; 3D view; checkCIF report

## Figures and Tables

**Table 1:** Hydrogen-bond geometry (Å, °)

*D*—H⋯*A*	*D*—H	H⋯*A*	*D*⋯*A*	*D*—H⋯*A*
N2—H2*N*⋯N3^i^	0.865 (19)	2.316 (19)	2.9719 (15)	132.8 (16)
N2—H2*N*⋯S1	0.865 (19)	2.660 (19)	3.1116 (11)	113.8 (15)
N4—H4*N*⋯O1	0.830 (19)	1.929 (18)	2.6274 (14)	141.2 (17)
N5—H5*N*⋯S1^ii^	0.856 (18)	2.576 (19)	3.4119 (11)	165.7 (16)

Symmetry codes: (i) 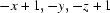; (ii) 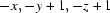.

**Fig. 1.**
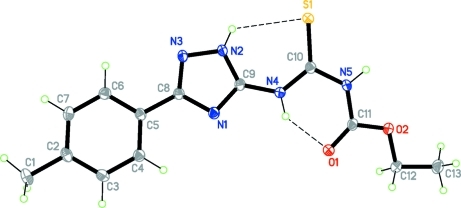
The molecular structure of I with the atomic numbering scheme. Displacement ellipsoids are drawn at the 50% probability level.

**Fig. 2.**
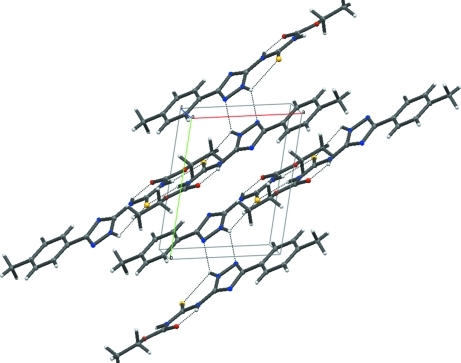
Crystal packing in the cell viewed along the axis c.

**Fig. 3.**
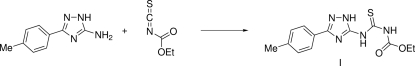
Synthesis of N-carbethoxy-N'-[3-(4-methylphenyl)-1H-1,2,4-triazol-5- yl]thiourea.

**Fig. 4.**
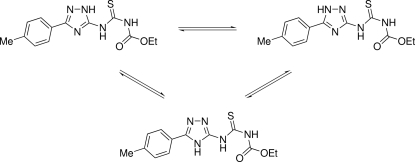
Annular tautomerism in N-carbethoxy-N'-[3(5)-(4-methylphenyl)-1(4)H-1,2,4-\ triazol-5(3)-yl]thiourea.

## supplementary crystallographic information

### Comment

Recently, we reported the crystal structure of *N*-carbethoxy-*N'*-(3-phenyl-1*H*-1,2,4-triazol-5-yl)thiourea (Dolzhenko *et al.*, 2010). Herein, in continuation of our investigations on annular tautomerism of 1,2,4-triazoles (Figs 3 and 4) in solutions (Dolzhenko *et al.*, 2009*a*) and crystalline state (Dolzhenko *et al.*, 2009*b*,*c*, 2010), we study the similar compound with methyl group presented in *para*-position of the phenyl ring. The electron donating effect of the methyl group might shift the tautomeric equilibrium towards the 5-aryl-3-thioureido-1*H*-1,2,4-triazole tautomeric form (Buzykin *et al.*, 2006; Dolzhenko *et al.*, 2009*a*). However, we found that this effect is not sufficient to alter the structure. Analogously to *N*-carbethoxy-*N'*-(3-phenyl-1*H*-1,2,4-triazol-5-yl)thiourea (Dolzhenko *et al.*, 2010), the title compound crystallizes with similar molecular structure (Fig. 1) and packing (Fig. 2). The N2—H···S1 hydrogen bonding between the endocyclic N(3)H proton of the triazole ring and the thioureido sulfur S1 atom (Fig.1 and Table 1) arranged in a *S*(6) graph-set motif (Bernstein *et al.*, 1995) is believed to be an essential factor stabilizing the tautomer. The triazole ring is planar with an r.m.s. deviation of 0.0069 Å. It makes a dihedral angle of 6.59 (10)° with the phenyl ring. The C10—N4 bond is significantly shorter (1.3414 (16) Å) than other C—N bonds of the carbethoxythiourea group (1.384–1.385 Å). Similarly to the previously reported related structures (Dolzhenko *et al.*, 2010; Huang *et al.*, 2009; Lin *et al.*, 2007; Lin *et al.*, 2004; Su *et al.*, 2006; Zhang *et al.*, 2007; Zhang *et al.*, 2003), the carbethoxythiourea group of the title compound adopts (*Z*)-configuration across the thiourea C10—N4 bond and (*E*)-configuration across the C11—N5 bond. The strong intramolecular N4—H···O1═C11 hydrogen bonding arranged in common for carbethoxythioureas (Dolzhenko *et al.*, 2010; Huang *et al.*, 2009; Lin *et al.*, 2007; Lin *et al.*, 2004; Su *et al.*, 2006; Zhang *et al.*, 2007; Zhang *et al.*, 2003) *S*(6) graph-set motif stabilizes this configuration.

In the crystal, the molecules form two types of centrosymmetric dimmers (Fig. 2, Table 1). The N3—N2H sides of two molecules are connected by intermolecular hydrogen bonds making the *R*^2^_2_(6) graph-set motif. Atom N5 is also involved in the intermolecular N—H···S interaction with the thiocarbonyl atom S1 of adjacent molecule making another pair with the *R*^2^_2_(8) graph-set motif similar to that observed in other carbethoxythioureas (Dolzhenko *et al.*, 2010; Huang *et al.*, 2009; Lin *et al.*, 2007; Lin *et al.*, 2004; Su *et al.*, 2006; Zhang *et al.*, 2007; Zhang *et al.*, 2003). Together, these hydrogen bonds connect molecules in a network parallel to the (111) plane.

### Experimental

The title compound (I) was synthesized by nucleophilic addition of 3(5)-amino-5(3)-(4-methylphenyl)-1*H*-1,2,4-triazole (Dolzhenko *et al.*, 2009*a*) to ethoxycarbonyl isothiocyanate in DMF solution at room temperature (Fig. 3). Single crystals suitable for crystallographic analysis were grown by recrystallization from ethanol.

### Refinement

All the H atoms attached to the carbon atoms were constrained in a riding motion approximation [0.95 Å for C_aryl_—H, 0.99 Å for methylenic protons and 0.98 Å for methyl group protons; U_iso_(H) = 1.2U_eq_(C_aryl_), U_iso_(H) = 1.2U_eq_(C_methylenic_) and U_iso_(H) = 1.5U_eq_(C_methyl_)] while the N-bound H atoms were located in a difference map and refined freely.

### Crystal data

**Table:**

C_13_H_15_N_5_O_2_S	*Z* = 2
*M**_r_* = 305.36	*F*(000) = 320
Triclinic, *P*1	*D*_x_ = 1.425 Mg m^−^^3^
Hall symbol: -P 1	Melting point: 489 K
*a* = 6.8430 (5) Å	Mo *K*α radiation, λ = 0.71073 Å
*b* = 8.7789 (6) Å	Cell parameters from 6864 reflections
*c* = 12.2563 (9) Å	θ = 2.4–27.5°
α = 90.780 (1)°	µ = 0.24 mm^−^^1^
β = 99.425 (1)°	*T* = 100 K
γ = 101.279 (1)°	Block, colourless
*V* = 711.52 (9) Å^3^	0.56 × 0.46 × 0.24 mm

### Data collection

**Table:**

Bruker SMART APEX CCD diffractometer	3243 independent reflections
Radiation source: fine-focus sealed tube	3088 reflections with *I* > 2σ(*I*)
graphite	*R*_int_ = 0.021
φ and ω scans	θ_max_ = 27.5°, θ_min_ = 1.7°
Absorption correction: multi-scan (*SADABS*; Sheldrick, 2001)	*h* = −8→8
*T*_min_ = 0.877, *T*_max_ = 0.945	*k* = −11→11
9015 measured reflections	*l* = −15→15

### Refinement

**Table:**

Refinement on *F*^2^	Primary atom site location: structure-invariant direct methods
Least-squares matrix: full	Secondary atom site location: difference Fourier map
*R*[*F*^2^ > 2σ(*F*^2^)] = 0.036	Hydrogen site location: inferred from neighbouring sites
*wR*(*F*^2^) = 0.095	H atoms treated by a mixture of independent and constrained refinement
*S* = 1.07	*w* = 1/[σ^2^(*F*_o_^2^) + (0.0529*P*)^2^ + 0.328*P*] where *P* = (*F*_o_^2^ + 2*F*_c_^2^)/3
3243 reflections	(Δ/σ)_max_ = 0.001
204 parameters	Δρ_max_ = 0.44 e Å^−^^3^
0 restraints	Δρ_min_ = −0.31 e Å^−^^3^

### Special details

**Table:**

Geometry. All esds (except the esd in the dihedral angle between two l.s. planes) are estimated using the full covariance matrix. The cell esds are taken into account individually in the estimation of esds in distances, angles and torsion angles; correlations between esds in cell parameters are only used when they are defined by crystal symmetry. An approximate (isotropic) treatment of cell esds is used for estimating esds involving l.s. planes.
Refinement. Refinement of *F*^2^ against ALL reflections. The weighted *R*-factor *wR* and goodness of fit *S* are based on *F*^2^, conventional *R*-factors *R* are based on *F*, with *F* set to zero for negative *F*^2^. The threshold expression of *F*^2^ > 2σ(*F*^2^) is used only for calculating *R*-factors(gt) *etc*. and is not relevant to the choice of reflections for refinement. *R*-factors based on *F*^2^ are statistically about twice as large as those based on *F*, and *R*- factors based on ALL data will be even larger.

### Fractional atomic coordinates and isotropic or equivalent isotropic displacement parameters (Å^2^)

**Table:**

	*x*	*y*	*z*	*U*_iso_*/*U*_eq_	
S1	0.23286 (5)	0.36065 (4)	0.52820 (2)	0.01721 (11)	
O1	0.17169 (14)	0.49382 (11)	0.16661 (8)	0.0193 (2)	
O2	−0.10045 (14)	0.57606 (10)	0.20980 (8)	0.0174 (2)	
N1	0.62121 (16)	0.25023 (12)	0.28575 (9)	0.0155 (2)	
N2	0.51358 (16)	0.16832 (13)	0.43785 (9)	0.0162 (2)	
H2N	0.440 (3)	0.145 (2)	0.4885 (16)	0.029 (5)*	
N3	0.65581 (16)	0.08284 (13)	0.42368 (9)	0.0173 (2)	
N4	0.35689 (16)	0.35967 (12)	0.33186 (9)	0.0156 (2)	
H4N	0.343 (3)	0.390 (2)	0.2676 (16)	0.024 (4)*	
N5	0.09899 (17)	0.48816 (13)	0.34439 (9)	0.0163 (2)	
H5N	0.016 (3)	0.512 (2)	0.3841 (15)	0.022 (4)*	
C1	1.3339 (2)	−0.07535 (17)	0.13315 (12)	0.0239 (3)	
H1A	1.4199	−0.1231	0.1888	0.036*	
H1B	1.4169	0.0132	0.1036	0.036*	
H1C	1.2688	−0.1521	0.0728	0.036*	
C2	1.17417 (19)	−0.02005 (15)	0.18576 (11)	0.0180 (3)	
C3	1.0630 (2)	0.08361 (15)	0.13251 (11)	0.0184 (3)	
H3	1.0898	0.1204	0.0628	0.022*	
C4	0.91420 (19)	0.13361 (14)	0.17991 (11)	0.0169 (3)	
H4	0.8391	0.2029	0.1420	0.020*	
C5	0.87416 (18)	0.08280 (14)	0.28277 (10)	0.0149 (2)	
C6	0.98549 (19)	−0.01994 (14)	0.33704 (11)	0.0169 (3)	
H6	0.9604	−0.0554	0.4073	0.020*	
C7	1.13279 (19)	−0.07028 (15)	0.28828 (11)	0.0180 (3)	
H7	1.2069	−0.1405	0.3258	0.022*	
C8	0.71732 (18)	0.13779 (14)	0.33214 (10)	0.0144 (2)	
C9	0.49501 (18)	0.26348 (14)	0.35425 (10)	0.0143 (2)	
C10	0.23405 (18)	0.40227 (14)	0.39616 (10)	0.0144 (2)	
C11	0.06534 (19)	0.51753 (14)	0.23292 (11)	0.0156 (2)	
C12	−0.1632 (2)	0.59864 (16)	0.09263 (11)	0.0197 (3)	
H12A	−0.2036	0.4972	0.0505	0.024*	
H12B	−0.0510	0.6633	0.0622	0.024*	
C13	−0.3400 (2)	0.67920 (17)	0.08463 (12)	0.0234 (3)	
H13A	−0.4464	0.6170	0.1192	0.035*	
H13B	−0.3926	0.6911	0.0066	0.035*	
H13C	−0.2958	0.7819	0.1229	0.035*	

### Atomic displacement parameters (Å^2^)

**Table:**

	*U*^11^	*U*^22^	*U*^33^	*U*^12^	*U*^13^	*U*^23^
S1	0.02084 (18)	0.01893 (17)	0.01474 (17)	0.00843 (12)	0.00598 (12)	0.00253 (11)
O1	0.0225 (5)	0.0213 (5)	0.0182 (4)	0.0100 (4)	0.0082 (4)	0.0051 (4)
O2	0.0192 (4)	0.0177 (4)	0.0178 (4)	0.0082 (3)	0.0049 (3)	0.0034 (3)
N1	0.0169 (5)	0.0127 (5)	0.0181 (5)	0.0040 (4)	0.0049 (4)	0.0016 (4)
N2	0.0162 (5)	0.0182 (5)	0.0172 (5)	0.0076 (4)	0.0066 (4)	0.0035 (4)
N3	0.0169 (5)	0.0190 (5)	0.0195 (5)	0.0089 (4)	0.0067 (4)	0.0042 (4)
N4	0.0183 (5)	0.0161 (5)	0.0147 (5)	0.0070 (4)	0.0051 (4)	0.0029 (4)
N5	0.0187 (5)	0.0170 (5)	0.0166 (5)	0.0089 (4)	0.0067 (4)	0.0019 (4)
C1	0.0195 (6)	0.0250 (7)	0.0298 (7)	0.0071 (5)	0.0087 (5)	−0.0045 (6)
C2	0.0151 (6)	0.0157 (6)	0.0232 (6)	0.0021 (5)	0.0050 (5)	−0.0045 (5)
C3	0.0209 (6)	0.0161 (6)	0.0195 (6)	0.0026 (5)	0.0082 (5)	0.0005 (5)
C4	0.0179 (6)	0.0143 (6)	0.0197 (6)	0.0044 (5)	0.0050 (5)	0.0020 (5)
C5	0.0147 (6)	0.0127 (5)	0.0176 (6)	0.0023 (4)	0.0043 (5)	−0.0010 (4)
C6	0.0182 (6)	0.0155 (6)	0.0172 (6)	0.0037 (5)	0.0032 (5)	0.0013 (5)
C7	0.0167 (6)	0.0148 (6)	0.0225 (6)	0.0054 (5)	0.0011 (5)	−0.0004 (5)
C8	0.0142 (5)	0.0130 (5)	0.0161 (6)	0.0028 (4)	0.0026 (4)	0.0002 (4)
C9	0.0143 (5)	0.0122 (5)	0.0165 (6)	0.0029 (4)	0.0027 (4)	−0.0005 (4)
C10	0.0153 (6)	0.0114 (5)	0.0167 (6)	0.0025 (4)	0.0036 (4)	0.0000 (4)
C11	0.0181 (6)	0.0109 (5)	0.0186 (6)	0.0038 (4)	0.0043 (5)	0.0014 (4)
C12	0.0219 (6)	0.0217 (6)	0.0176 (6)	0.0091 (5)	0.0034 (5)	0.0042 (5)
C13	0.0197 (6)	0.0241 (7)	0.0286 (7)	0.0090 (5)	0.0048 (5)	0.0060 (5)

### Geometric parameters (Å, °)

**Table:**

S1—C10	1.6649 (13)	C1—H1C	0.9800
O1—C11	1.2173 (16)	C2—C7	1.3915 (19)
O2—C11	1.3260 (15)	C2—C3	1.3988 (18)
O2—C12	1.4578 (15)	C3—C4	1.3889 (17)
N1—C9	1.3184 (16)	C3—H3	0.9500
N1—C8	1.3701 (15)	C4—C5	1.3943 (17)
N2—C9	1.3364 (16)	C4—H4	0.9500
N2—N3	1.3704 (14)	C5—C6	1.3984 (17)
N2—H2N	0.865 (19)	C5—C8	1.4691 (17)
N3—C8	1.3261 (16)	C6—C7	1.3903 (17)
N4—C10	1.3414 (16)	C6—H6	0.9500
N4—C9	1.3839 (15)	C7—H7	0.9500
N4—H4N	0.830 (19)	C12—C13	1.5067 (18)
N5—C11	1.3840 (16)	C12—H12A	0.9900
N5—C10	1.3853 (16)	C12—H12B	0.9900
N5—H5N	0.856 (18)	C13—H13A	0.9800
C1—C2	1.5088 (17)	C13—H13B	0.9800
C1—H1A	0.9800	C13—H13C	0.9800
C1—H1B	0.9800		
			
C11—O2—C12	114.69 (10)	C7—C6—C5	120.07 (12)
C9—N1—C8	102.36 (10)	C7—C6—H6	120.0
C9—N2—N3	109.09 (10)	C5—C6—H6	120.0
C9—N2—H2N	130.3 (12)	C6—C7—C2	121.48 (12)
N3—N2—H2N	119.7 (12)	C6—C7—H7	119.3
C8—N3—N2	102.49 (10)	C2—C7—H7	119.3
C10—N4—C9	129.41 (11)	N3—C8—N1	114.56 (11)
C10—N4—H4N	116.0 (12)	N3—C8—C5	123.37 (11)
C9—N4—H4N	114.5 (12)	N1—C8—C5	122.06 (11)
C11—N5—C10	126.49 (11)	N1—C9—N2	111.46 (11)
C11—N5—H5N	117.7 (12)	N1—C9—N4	120.65 (11)
C10—N5—H5N	115.0 (12)	N2—C9—N4	127.77 (11)
C2—C1—H1A	109.5	N4—C10—N5	114.73 (11)
C2—C1—H1B	109.5	N4—C10—S1	125.77 (9)
H1A—C1—H1B	109.5	N5—C10—S1	119.49 (9)
C2—C1—H1C	109.5	O1—C11—O2	125.35 (12)
H1A—C1—H1C	109.5	O1—C11—N5	125.32 (12)
H1B—C1—H1C	109.5	O2—C11—N5	109.33 (11)
C7—C2—C3	118.02 (11)	O2—C12—C13	106.66 (11)
C7—C2—C1	121.23 (12)	O2—C12—H12A	110.4
C3—C2—C1	120.75 (12)	C13—C12—H12A	110.4
C4—C3—C2	121.03 (12)	O2—C12—H12B	110.4
C4—C3—H3	119.5	C13—C12—H12B	110.4
C2—C3—H3	119.5	H12A—C12—H12B	108.6
C3—C4—C5	120.49 (12)	C12—C13—H13A	109.5
C3—C4—H4	119.8	C12—C13—H13B	109.5
C5—C4—H4	119.8	H13A—C13—H13B	109.5
C4—C5—C6	118.90 (11)	C12—C13—H13C	109.5
C4—C5—C8	119.80 (11)	H13A—C13—H13C	109.5
C6—C5—C8	121.30 (11)	H13B—C13—H13C	109.5
			
C9—N2—N3—C8	1.76 (13)	C4—C5—C8—N1	−6.63 (18)
C7—C2—C3—C4	−0.71 (19)	C6—C5—C8—N1	173.24 (11)
C1—C2—C3—C4	179.27 (12)	C8—N1—C9—N2	0.94 (14)
C2—C3—C4—C5	0.84 (19)	C8—N1—C9—N4	−175.31 (11)
C3—C4—C5—C6	−0.36 (19)	N3—N2—C9—N1	−1.78 (15)
C3—C4—C5—C8	179.52 (11)	N3—N2—C9—N4	174.14 (12)
C4—C5—C6—C7	−0.23 (19)	C10—N4—C9—N1	−171.44 (12)
C8—C5—C6—C7	179.89 (11)	C10—N4—C9—N2	13.0 (2)
C5—C6—C7—C2	0.36 (19)	C9—N4—C10—N5	−173.84 (12)
C3—C2—C7—C6	0.11 (19)	C9—N4—C10—S1	6.13 (19)
C1—C2—C7—C6	−179.87 (12)	C11—N5—C10—N4	7.69 (18)
N2—N3—C8—N1	−1.24 (14)	C11—N5—C10—S1	−172.28 (10)
N2—N3—C8—C5	179.33 (11)	C12—O2—C11—O1	5.70 (18)
C9—N1—C8—N3	0.24 (14)	C12—O2—C11—N5	−174.54 (10)
C9—N1—C8—C5	179.68 (11)	C10—N5—C11—O1	−12.6 (2)
C4—C5—C8—N3	172.75 (12)	C10—N5—C11—O2	167.62 (11)
C6—C5—C8—N3	−7.38 (19)	C11—O2—C12—C13	−174.72 (11)

### Hydrogen-bond geometry (Å, °)

**Table:**

*D*—H···*A*	*D*—H	H···*A*	*D*···*A*	*D*—H···*A*
N2—H2N···N3^i^	0.865 (19)	2.316 (19)	2.9719 (15)	132.8 (16)
N2—H2N···S1	0.865 (19)	2.660 (19)	3.1116 (11)	113.8 (15)
N4—H4N···O1	0.830 (19)	1.929 (18)	2.6274 (14)	141.2 (17)
N5—H5N···S1^ii^	0.856 (18)	2.576 (19)	3.4119 (11)	165.7 (16)

Symmetry codes: (i) −*x*+1, −*y*, −*z*+1; (ii) −*x*, −*y*+1, −*z*+1.
